# Sandwich-Type Nitrogen and Sulfur Codoped Graphene-Backboned Porous Carbon Coated Separator for High Performance Lithium-Sulfur Batteries

**DOI:** 10.3390/nano8040191

**Published:** 2018-03-26

**Authors:** Feng Chen, Lulu Ma, Jiangang Ren, Xinyu Luo, Bibo Liu, Xiangyang Zhou

**Affiliations:** 1 School of Resource and Environment, Henan University of Engineering, No. 1, Xianghe Road, Zhengzhou 451191, China; chenfeng871588@163.com (F.C.); malulu1001@163.com (L.M.); renjiangang2005@126.com (J.R.); 2 School of Metallurgy and Environment, Central South University, Lushan South Street 932, Yuelu District, Changsha 410083, China; 17307484092@163.com

**Keywords:** heteroatom doping, multifunctional separators, shuttle effect, graphene-backboned porous carbon, lithium-sulfur batteries

## Abstract

Lithium-sulfur (Li-S) batteries have been identified as the greatest potential next- generation energy-storage systems because of the large theoretical energy density of 2600 Wh kg^−1^. However, its practical application on a massive scale is impeded by severe capacity loss resulted from the notorious polysulfides shuttle. Here, we first present a novel technique to synthesize sandwich-type nitrogen and sulfur codoped graphene-backboned porous carbon (NSGPC) to modify the commercial polypropylene separator in Li-S batteries. The as-synthesized NSGPC exhibits a unique micro/mesoporous carbon framework, large specific surface area (2439.0 m^2^ g^−1^), high pore volume (1.78 cm^3^ g^−1^), good conductivity, and in situ nitrogen (1.86 at %) and sulfur (5.26 at %) co-doping. Benefiting from the particular physical properties and chemical components of NSGPC, the resultant NSGPC-coated separator not only can facilitate rapid Li^+^ ions and electrons transfer, but also can restrict the dissolution of polysulfides to alleviate the shuttle effect by combining the physical absorption and strong chemical adsorption. As a result, Li-S batteries with NSGPC-coated separator exhibit high initial reversible capacity (1208.6 mAh g^−1^ at 0.2 C), excellent rate capability (596.6 mAh g^−1^ at 5 C), and superior cycling stability (over 500 cycles at 2 C with 0.074% capacity decay each cycle). Propelling our easy-designed pure sulfur cathode to a extremely increased mass loading of 3.4 mg cm^−2^ (70 wt. % sulfur), the Li-S batteries with this functional composite separator exhibit a superior high initial capacity of 1171.7 mAh g^−1^, which is quite beneficial to commercialized applications.

## 1. Introduction

In recent years, lithium-sulfur (Li-S) batteries have been identified as the greatest potential next- generation energy-storage devices because of their large theoretical energy density (2600 Wh kg^−1^) and theoretical specific capacity (1675 mAh g^−1^). Furthermore, sulfur has some obvious advantages over current transition metals cathodes in lithium-ion batteries of natural abundance, inexpensive and eco-friendliness [1,2]. Despite their appealing features, the practical application of Li-S batteries is still seriously impeded by several drawbacks, including: (i) the poor electrical conductivity of sulfur and its final discharge product Li_2_S/Li_2_S_2_, leading to sluggish electron transfer and low sulfur utilization; (ii) the severe volume expansion (~80%) caused by sulfur reduction to Li_2_S, resulting in capacity decaying and structural instability; and (iii) the dissolution and notorious “shuttle effect” of lithium polysulfides, giving rise to poor coulombic efficiency and inferior cycling life [3,4]. To tackle these issues, extensive strategies have been explored to fabricate different structures of host materials for confining sulfur, including various porous carbon materials, graphene, carbon nanotube, conducting polymers, metal oxides/sulfides, metal-organic frameworks, and covalent-organic frameworks [5,6,7,8,9,10,11,12,13,14]. However, these sulfur cathode composites generally involve complicated and time-consuming processing steps, consumption or generation of some deleterious substances, and low sulfur content or sulfur mass loading in the electrode, which significantly limit the extensive commercial applications of Li-S batteries. 

Alternatively, designing a novel Li-S cell configuration with a coated separator or a free- standing interlayer has been proven to be another effective way to address the issues described above [15,16]. As a critical component in a battery, the separator is a porous polymer membrane, which has a primary function of maintaining the free ionic pathways and preventing the internal short circuit. However, the conventional polyolefin separators employed in lithium-ion batteries, such as polyethylene and polypropylene (PP) membranes, are not capable of suppressing polysulfides shuttle and stabilizing lithium anode on account of the large-pore framework (about 10^2^ nm) [17]. Therefore, all sorts of conductive carbon materials, such as commercial Super P, multiwalled carbon nanotube, carbon nanofiber, graphene, as well as microporous, mesoporous and macroporous carbons [18,19,20,21,22,23,24], have all been put forward to modify the separator or as the interlayer to physically confine the polysulfides in the cathode area, keeping the active sulfur approachable to further reutilization during the following cycles. Furthermore, the coating layer or interlayer can serve as an upper current collector to promote the electron transfer during the redox reaction [23,24]. Unfortunately, the weak physical adsorption between nonpolar carbon and polar polysulfides leads to problems in long-term cycling [25]. Consequently, strong chemical binding of lithium polysulfides to the carbon hosts is badly needed to improve polysulfides adsorption and, thus, active material utilization and cycling life of Li-S batteries.

Latterly, the chemical functionalization of carbon-based materials via heteroatom doping (such as N, P, B, and S) was widely reported to offer such strong chemical interactions with lithium polysulfides [6,17,26,27]. For example, N-doped porous carbons or N-doped graphene reveal substantial chemical adsorption of lithium polysulfides attributed to the strong Li-N interactions between Li^+^ cations in Li_2_S_x_ and electronegative N atoms in the carbon material, related to the electron donating property of N element [28,29,30]. Apart from sole N doping, our research team and other studies have discovered that S doping could improve the electric conductivity of carbon, raise the appetency between polysulfides and S-doped carbon materials, and facilitate better anchor polysulfides to enhance the electrochemical properties of Li-S batteries [27,31]. Very recently, double heteroatom doping has started to obtain attention in the field of Li-S batteries. S-encapsulated carbon matrices with N and S dual-doping, such as, polyrhodanine@cellulose-derived N and S dual-doped carbon, N/S-codoped graphene sponge, coral-like N and S co-doped mesoporous carbon, and N and S dual-doped hierarchical porous biomass carbon [32,33,34,35], have shown stronger polysulfides chemisorption compared to single N doped and undoped carbons. Moreover, the Ab initio calculations according to density functional theory (DFT) highly confirm these experimental results [32,33]. However, little work has been conducted on the utilization of N and S dual-doped carbon materials to modify separator for Li-S batteries [36,37]. Therefore, the N and S dual-doped carbon materials coated separator would be a promising approach to repress the polysulfides shuttle and to enhance the performance of Li-S batteries.

Herein, we present a new technology to fabricate sandwich-type nitrogen and sulfur codoped graphene-backboned porous carbon (NSGPC) coated PP separator for use in Li-S batteries. The NSGPC was synthesized through a facile hydrothermal process using graphene oxide (GO) and sucrose, thiourea as the carbon precursors and heteroatom source, followed by a KOH activation step. In NSGPC, the graphene nanosheets served as the scaffold, guaranteeing the extraordinary electronic conductivity. Then the micro/mesoporous carbon was grown on graphene nanosheets by in situ N and S co-doping, not only preventing the agglomerates of graphene nanosheets, but also bringing about a higher specific surface area and enhanced electrical conductivity [38]. Subsequently, the NSGPC was fabricated to coat on one side of a commercial PP separator, forming a NSGPC-coated separator. As far as we know, the modification of PP separator with this sandwich-type NSGPC has not been reported so far. In addition, the cell configuration suggested by this NSGPC-coated separator allows the preparation of pure sulfur cathode via the conventional slurry-coating method, which simplifies the synthesis procedure and provides enormous potential for significantly increasing the content and mass loading of the sulfur electrode. Benefiting from the special structural characteristics and in situ N and S co-doping, the resultant NSGPC-coated separator could effectively address the polysulfides shuttle by combining the physical absorption and strong chemical adsorption, and then enhance the electrochemical properties of Li-S batteries in the aspect of high reversible capacity and excellent rate capability as well as long-term cycling life.

## 2. Materials and Methods

### 2.1. Materials Synthesis

#### 2.1.1. Preparation of Sandwich-Type Nitrogen and Sulfur Codoped Graphene-Backboned Porous Carbon (NSGPC)

The NSGPC was synthesized by a facile hydrothermal process using graphene oxide (GO) and sucrose, thiourea as the carbon precursors and heteroatom source, followed by a KOH activation step. Typically, graphene oxide (GO) was first prepared by a modified Hummer’s method [39]. 0.35 g of thiourea and 4.0 g of sucrose were slowly added into 67 mL of GO solution (3.0 mg mL^−1^) and sonicated for 1 h to form a homogenous mixture. After keeping magnetic stirring for 4 h, the mixture was then transferred into a Teflon-lined stainless steel autoclave (100 mL) and kept at 180 °C for 12 h. The autoclave was allowed to cool naturally, the resulting solid product was washed with deionized water completely and dried at 60 °C in a vacuum oven for 12 h. Then, this intermediate product (2 g) was mixed with KOH (8 g) and placed in a horizontal tube furnace, heated to 800 °C with a heating rate of 5 °C min^−1^ and kept for 2 h in an Ar atmosphere. After cooling to ambient temperature, the obtained product was washed with 0.5 M hydrochloric acid solution and distilled water until the pH value reached 7. The final NSGPC was obtained after dried in a drying oven at 110 °C for 24 h. The pure graphene was also prepared as a control sample using a similar hydrothermal process without adding sucrose and thiourea.

#### 2.1.2. Preparation of NSGPC-Coated Separator

The NSGPC-coated separator was prepared via a slurry-coating method. The NSGPC (90 wt. %) and polyvinylidene fluoride (PVDF) (10 wt. %) were grounded in a mortar with *N*-methyl-2-pyrrolidinone (NMP) as the dispersant for 0.5 h, then the slurry was evenly coated on one side of the commercial PP separator (Celgard 2400, Hefei kejing material technology co. LTD., Hefei, China) with a scraper blade. The NSGPC-coated separator was obtained after dried overnight at 50 °C in a vacuum oven and then punched into small disks of 19 mm diameter. The mass loading of NSGPC on the separator was about 0.49 mg cm^−2^.

#### 2.1.3. Synthesis of Li_2_S_4_ and NSGPC-Li_2_S_4_ for Studying the Polysulfides Adsorption

The Li_2_S_4_ solution (0.02 M) was synthesized by stoichiometrically dissolving Li_2_S and sublimed S in a molar ratio of 1:3 in a mixture of 1,3-dioxolane (DOL) and dimethoxyethane (DME) (1:1 volume ratio) at 50 °C for 24 h under intense stirring, forming a homogeneous dark brown solution. The solvent was afterwards vaporized and the resultant solid matter was washed with toluene and dried in a vacuum, ultimately obtaining a yellow powder (Li_2_S_4_). Then, 80 mg NSGPC was added to 5 mL Li_2_S_4_ solution (0.02 M) to obtain the NSGPC-Li_2_S_4_ solution. The solution was permitted to stand for 0.5 h, and the precipitated solid was dried under vacuum to obtain NSGPC-Li_2_S_4_ for the XPS test. All these procedures were carried out in a glove box filled with Ar (Super 1220/750, MIKROUNA, Shanghai, China), in which water and oxygen contents were less than 0.1 ppm.

### 2.2. Materials Characterization

The crystalline structures of the above samples were characterized by an X-ray diffractometer (XRD, Rigaku-TTRIII, Tokyo, Japan) with Cu Kα (Cu-1.8 kW, λ = 0.154 nm) radiation. The surface morphologies were analyzed by scanning electron microscope (SEM, JSM-6360LV, Tokyo, Japan) and transmission electron microscopy (TEM, Titan G2 60-300, FEI, Hillsboro, OR, USA). An energy dispersive spectrometer (EDS) of the SEM and TEM apparatuses was used to analyze the elemental mapping. Raman spectra were recorded by a confocal Raman microscope (LabRAM Hr800, HORIBA Jobin Yvon, Tokyo, Japan) with an excitation wavelength of 532 nm. The Brunauer- Emmett-Teller (BET) tests were conducted using automatic specific surface area and porosity analyzer (ASAP 2020 HD88, Micromeritics, Norcross, GA, USA). The surface chemical states of the samples were analyzed by X-ray photoelectron spectroscopy (XPS, K-Alpha 1063, Thermo Fisher Scientific, Cambridge, MA, USA) measurements. A Canon EOS 750D Camera was used to obtain the digital photographs.

### 2.3. Electrochemical Measurements

The conventional slurry-coating approach was employed to prepare the pure sulfur cathode. For NSGPC-coated separator, the commercial sulfur powder, conductive carbon black, and PVDF binder were mixed in a weight ratio of 7:2:1 using NMP as the dispersant to form a homogeneous slurry. The slurry was uniformly spread onto aluminum foils with a scraper blade, and vacuum dried at 50 °C for 12 h, and then punched into small disks with a diameter of 10 mm. Depending on the electrochemical tests, the mass loading of the sulfur cathodes was about 1.3 or 3.4 mg cm^−2^. In contrast, conventional Li-S batteries with original PP separator and pure sulfur cathode were measured using a 50 wt. % sulfur electrode, which is equivalent to the sulfur ratio in the cathode when the weight of NSGPC coating is counted into the 70 wt. % sulfur cathode, and the sulfur mass loading was about 1.3 mg cm^−2^. 

The standard CR2025-type stainless steel button cells were assembled in an Ar-filled glove box. The pure sulfur electrode was used as the cathode, lithium foil was used as the anode, and pristine PP separator and NSGPC-coated separator were used as the separators. The electrolyte was composed of 1.0 M lithium bis-(trifluoromethanesulfonyl)imide (LiTFSI) salt and 0.1 M LiNO_3_ in a solvent mixture of 1,3-dioxolane (DOL) and dimethoxyethane (DME) (1:1 volume ratio). The cells were discharged and charged over the voltage range 1.7–2.8 V at different current densities of 0.2–5 C (1 C = 1675 mA g^−1^) using a battery test system (LAND CT-2001A, Wuhan LAND ekectronics Limited by Share, Wuhan, China). In this paper, the specific capacities were calculated based on the mass of sulfur. The cyclic voltammogram (CV) test was performed at a scanning rate of 0.2 mV S^−1^ with a wide potential range of 1.6–3.0 V on an electrochemical workstation (PARSTAT 4000, AMETEK, San Diego, CA, USA). The electrochemical impedance spectroscopy (EIS) was also carried out using the same instrument from 100 kHz to 10 mHz, the corresponding applied voltage is 10 mV.

## 3. Results and Discussion

**Figure 1:**
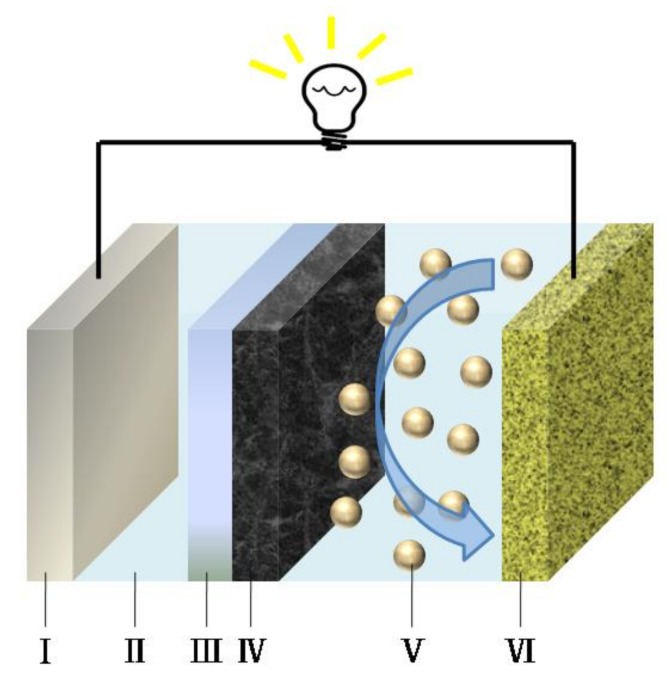
The schematic of a Li-S cell configuration with a NSGPC-coated separator: I. Li anode, II. electrolyte, III. pristine PP separator, IV. NSGPC coating, V. polysulfides, VI. sulfur cathode.

The schematic illustration of Li-S cells with NSGPC-coated separator is shown in Figure 1, where the NSGPC coating is faced toward the sulfur cathode, acting to restrain the migrating polysulfides through physical absorption and strong chemical adsorption, and serving as a conductive upper current collector to reutilize the intercepted active materials during the following cycles. Thus, the resulting NSGPC-coated separator is expected to significantly enhance the electrochemical property of Li-S batteries.

**Figure 2:**
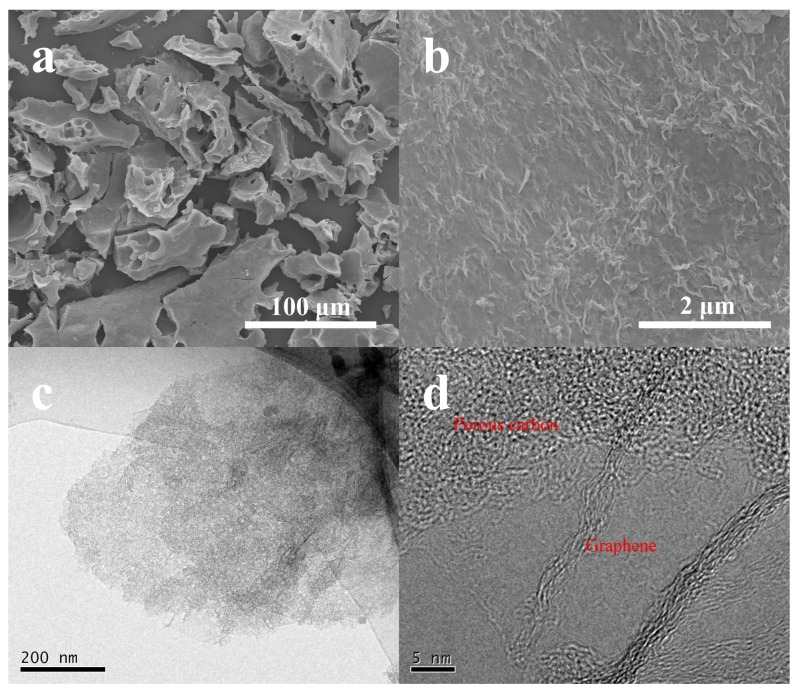
SEM images of NSGPC (**a**,**b**), TEM and HRTEM images of NSGPC (**c**,**d**).

The SEM and TEM were first employed to characterize the morphologies and structures of the as-obtained pure graphene (G) and NSGPC. SEM images as shown in Figure S1a,b reveal that the pure G has a well-defined sheet-like structure with some wrinkles on the surface, which is characteristic of graphene sheets [40]. Compared with the pure G, the NSGPC still remains as the lamella-like structure with a dimension of several tens to hundreds of microns (Figure 2a). Besides, it can be seen that some holes with different sizes (mesopores and macropores) are discovered on the surface of NSGPC, which is caused by KOH activation. Moreover, as shown in Figure 2b, plentiful small curvatures and wrinkles exist on the surface of NSGPC, strikingly distinguishing from the morphology of pure G, which could be due to the growth of sucrose-derived amorphous carbon on graphene nanosheets. 

TEM image of pure G testifies the transparent, thin, wrinkled and undular morphology (Figure S1c). It is suggested that scrolling and corrugation are part of the inherent nature of G, which stems from the truth that two dimension membrane structures tend to thermodynamically stable through bending [41]. Due to folding and scrolling of G, we would be capable of observing the cross-sectional profile of stacked graphene layers. The high-resolution TEM (HRTEM) image as displayed in Figure S1d demonstrates that the sheet is made up of less than ten layers of graphene. However, the TEM image (Figure 2c) of NSGPC shows a typical sheet-like structure and the surface of graphene nanosheets is clearly covered by amorphous carbon with abundant micro/mesopores. This indicated that we have fabricated a novel sandwich-type porous carbon material, in which amorphous carbon layers were grown on both sides of the graphene nanosheets. The HRTEM image of NSGPC in Figure 2d further confirms the presence of densely formed porous structures on the surface of the graphene nanosheets. 

Figure S2 shows the STEM image of NSGPC and the corresponding elemental mappings of C, O, N, and S, respectively, demonstrating the successfully incorporated N and S atoms into the carbon framework. The elemental mappings further confirm the evenly distribution of C, O, N, and S atoms throughout the NSGPC. This outcome suggests that the doped heteroatoms are evenly present both on the basal plane and on the edges of graphene nanosheets, as well as on the porous carbon layers. These nitrogen/sulfur-containing functional groups in the NSGPC are reported to be crucially important for long-term cycling life by effectively constraining the polysulfides shuttle through strong chemical adsorption [32,33,42].

**Figure 3:**
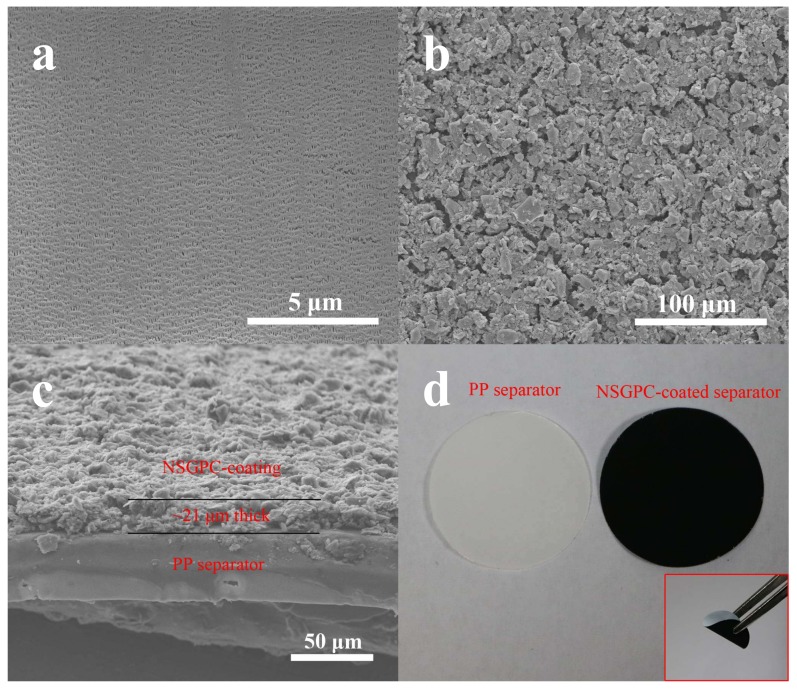
SEM images of the surface of pristine PP separator (**a**), the surface of NSGPC-coated separator (**b**), and the cross-section of NSGPC-coated separator (**c**), digital photograph of pristine PP separator and NSGPC-coated separator, with the inset indicating the flexibility of NSGPC-coated separator (**d**).

The surface morphologies of the pristine PP separator and NSGPC-coated separator were examined by SEM. As shown in Figure 3a, a plane surface with submicron pores all over the whole separator is observed for pristine PP separator. These long-narrow pores offer interconnected channels for polysulfides shuttle, which is the primary cause of the capacity fading of Li-S batteries with short cycle lifetime [16]. After the coating of NSGPC barrier layer, NSGPC is uniformly adhered to the routine PP separator and forms a compact carbon layer without discernible cracks (Figure 3b). As revealed in Figure 3c, we can see that the NSGPC layer was stacked well on the surface of PP separator, the NSGPC coating is about 21 μm in thickness and only 0.49 mg cm^−2^ in weight. Nevertheless, the thickness and weight of the routine PP separator are approximately 42 μm and 1.3 mg cm^−2^, respectively, which are much thicker and heavier than the NSGPC coating layer. The digital photograph of pristine PP separator and NSGPC-coated separator is presented in Figure 3d, it is obvious that a flexible NSGPC-coated separator which exhibits a exceptionally stable adhesion of the NSGPC to the surface of PP separator with good structure integrality even after bending back and forth many times.

**Figure 4:**
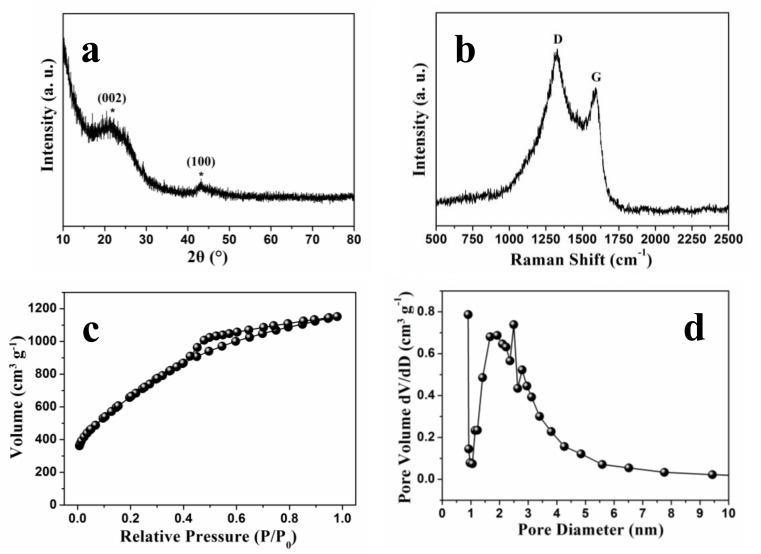
XRD pattern (**a**), Raman spectrum (**b**), N_2_ adsorption/desorption isotherm (**c**), and pore size distribution curve (**d**) of NSGPC.

The graphitization degree of NSGPC was described by the XRD pattern (Figure 4a) and Raman spectrum (Figure 4b). Two broad diffraction peaks located in the range from 20° to 30° and from 40° to 50° emerged in the XRD pattern are assigned to the (002) and (100) lattice planes, suggesting that the synthesized NSGPC has a low degree of crystallinity as previously reported in the literatures [31,32]. The Raman spectrum of NSGPC exhibits two prominent peaks located at 1328 cm^−1^ (D band) and 1591 cm^−1^ (G band), which are concerned with the disordered carbon and graphitized carbon, respectively [3,17]. The appearance of G band for NSGPC means its good electrical conductivity. Furthermore, it is generally believed that the intensity ratio of the D band to G band (I_D_/I_G_) can reflect the degree of defects in carbon materials [26]. The value of I_D_/I_G_ for NSGPC is calculated to be 1.17, manifesting that the presence of a lot of structural defects in NSGPC, which can be probably attributed to the KOH activation and the successful N and S doping. 

The N_2_ adsorption/desorption isotherm was employed to investigate the BET specific surface area and pore structure of the as-prepared NSGPC. As shown in Figure 4c, the NSGPC has certain nitrogen adsorption below the relative pressure of about 0.1 (P/P_0_), manifesting the existence of exclusive micropores [43]. Moreover, the distinctive hysteresis loop in the range of 0.4–0.9 P/P_0_ for NSGPC indicates a highly accessible pore geometry with very small mesoporous. According to the BET analysis, the specific surface area and total pore volume of NSGPC are calculated to be 2439.0 m^2^ g^−1^ and 1.78 cm^3^ g^−1^, respectively. A similar result was observed for the pore size distribution of NSGPC according to density functional theory (DFT). As shown in Figure 4d, the pore size ranges from about 0.5 to 10 nm, which further confirms the presence of characteristic micro/mesoporous hybrid structure in NSGPC. The average pore size of NSGPC is about 2.92 nm. Reports suggest that such a microporous/small-mesoporous carbon material not only can inhibit the dissolution of polysulfides through physical adsorption and thus promote the cycling reversibility of Li-S batteries, but also can facilitate electrolyte infiltration and provide rapid transport channels for Li^+^ ions and electrons, and thus reutilize the intercepted active sulfur [31,44].

**Figure 5:**
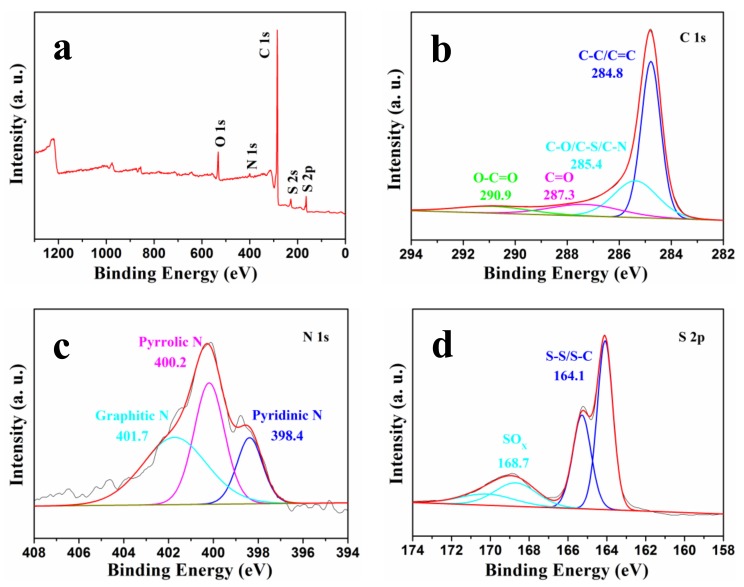
(**a**) XPS spectrum of NSGPC, high-resolution spectra of (**b**) C 1s, (**c**) N 1s, and (**d**) S 2p for NSGPC.

The surface chemical ingredients and functional groups of the NSGPC were identified by XPS (Figure 5). As displayed in Figure 5a, the XPS survey spectrum of NSGPC reveals four binding energy peaks for C 1s (284.8 eV), O 1s (531.2 eV), N 1s (400.3 eV), and S 2p (164.1 eV), showing the identical elemental component as that detected by EDS (Figure S2). As detected by XPS results, the C, O, N, and S contents in the NSGPC are 84.91, 7.97, 1.86, and 5.26 at %, respectively. The XPS results confirm the successful incorporation of both N and S atoms within the NSGPC structure. The high-resolution spectrum of C 1s in NSGPC (Figure 5b) can be deconvoluted into several single peaks, corresponding to C-C/C=C (284.8 eV), C-O/C-S/C-N (285.4 eV), C=O (287.3 eV), and O-C=O (290.9 eV) [32,33,34]. The existence of C-O/C-S/C-N species further demonstrates that N and S heteroatoms have been effectively doped into the carbon framework. The high-resolution N 1s spectrum of NSGPC (Figure 5c) shows three nitrogen species: graphitic N (401.7 eV), pyrrolic N (400.2 eV), and pyridinic N (398.4 eV), which are typically observed as for N-doped carbons. These nitrogen-functional groups can chemically adsorb polysulfides through interaction with pyrrolic N and pyridinic N atoms [30,37]. On the other hand, the S 2p spectrum of NSGPC (Figure 5d) can be deconvoluted into four peaks, which are equivalent to, respectively, S-S/S-C bonds at 164.1 and 165.3 eV, and SO_x_ species at 168.7 and 170.2 eV. These sulfur-containing functional groups are believed to increase the affinity of nonpolar carbon matrix to adsorb polar polysulfides [27,31,34].

**Figure 6:**
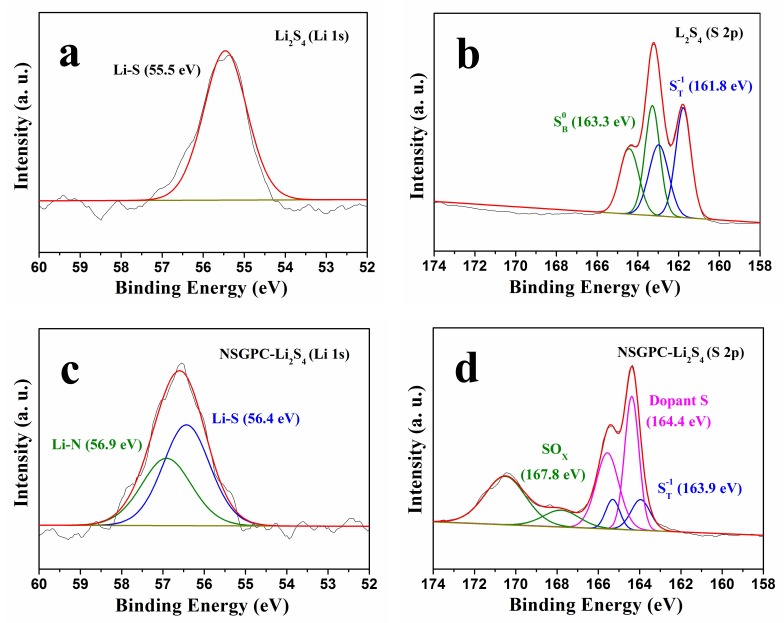
High-resolution XPS (**a**,**c**) Li 1s and (**b**,**d**) S 2p spectra of (**a**,**b**) Li_2_S_4_ and (**c**,**d**) NSGPC-Li_2_S_4_.

In order to experimentally illustrate the strong adsorption of NSGPC for polysulfides, a Li_2_S_4_ solution (0.02 M) was chosen as a representative of lithium polysulfides. Then, 80 mg NSGPC powder was added into 5 mL Li_2_S_4_ solution. Figure S3 shows the resulting digital photograph of the Li_2_S_4_ solution and after adding NSGPC. We can clearly see that the color of Li_2_S_4_ solution turns from the initially deep brown to light yellow after aging for 30 min, strongly suggesting that much of the Li_2_S_4_ has been absorbed by NSGPC. XPS tests were then performed to examine the types of interactions between NSGPC and Li_2_S_4_. We can see that only one symmetrical peak located at 55.5 eV is typically observed for the Li 1s spectrum of pristine Li_2_S_4_ (Figure 6a). However, when Li_2_S_4_ interacts with NSGPC, the Li 1s spectrum of NSGPC-Li_2_S_4_ displays an asymmetrical peak with higher binding energy (Figure 6c). The additional peak observed at 56.9 eV can be assigned to the Li^+^ cations interacting with doped N atoms in the NSGPC (Li-N) [28,29,30]. The high-resolution S 2p spectrum of Li_2_S_4_ exhibits two sulfur contributions at 161.8 and 163.3 eV with a 1:1 ratio, which was attributed to the terminal (S_T_^−1^) and bridging sulfur (S_B_^0^) atoms, respectively (Figure 6b) [32,45]. Compared to Li_2_S_4_ itself, the S 2p spectrum of NSGPC-Li_2_S_4_ was radically different, revealing disparate sulfur environments (Figure 6d). A large shift of 2.1 eV to the higher binding energy (at 163.9 and 165.4 eV) was observed for the S_T_^−1^, testifying the decrease of electron concentration on the S_T_^−1^, which might be resulted from the strong interaction between Li_2_S_4_ and doped S atoms in the NSGPC (S-S) [32]. The S_B_^0^ cannot be observed for NSGPC-Li_2_S_4_, because its binding energy zone overlaps with the dopant S. Above all, the XPS analysis provides overwhelming evidence of the strong chemical interaction between NSGPC and polysulfides, thus the resultant NSGPC-coated separator is expected to contribute to remarkably improve the electrochemical performance of Li-S batteries.

**Figure 7:**
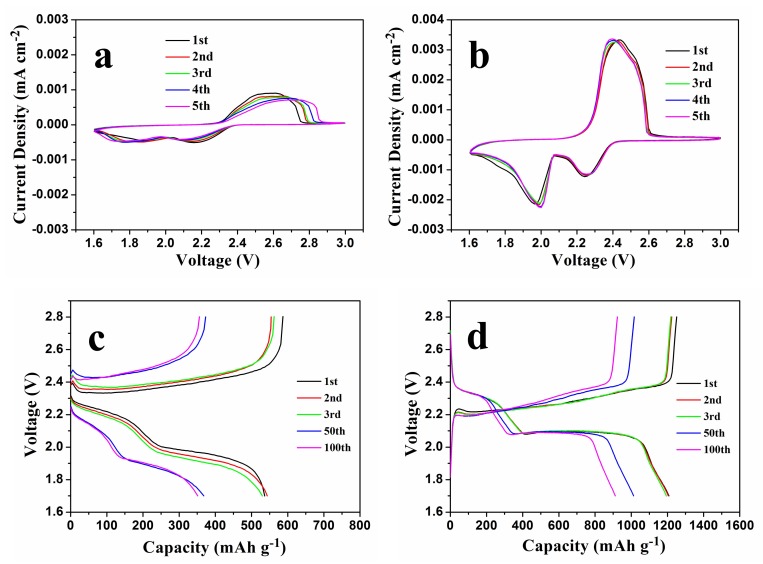
CV curves at a scan rate of 0.2 mV s^−1^ of the Li-S batteries with (**a**) PP and (**b**) NSGPC-coated separators, discharge/charge voltage profiles of the Li-S batteries with (**c**) PP and (**d**) NSGPC-coated separators at 0.2 C. The sulfur mass loading of the simple cathodes was about 1.3 mg cm^−2^.

To evaluate the potential advantages of NSGPC-coated separator for suppressing the polysulfides shuttle, the electrochemical performances of the Li-S batteries with PP and NSGPC-coated separators were evaluated in the CR2025-type button cells. The CV curves of the Li-S batteries with PP and NSGPC-coated separators are first displayed in Figure 7a,b, respectively. In the cathodic scan, there are two remarkable cathodic peaks, which can be allocated to the two-step reduction reaction from elemental sulfur to long-chain polysulfides (Li_2_S_x_, 4 ≤ x ≤ 8) and the further reduction to insoluble Li_2_S_2_ and finally to Li_2_S [3,4]. In the succeeding anodic scan, two partially overlapping oxidation peaks are observed, which probably due to the reversible conversion of Li_2_S_2_/Li_2_S to low-order polysulfides and then to high-order polysulfides, respectively [4,10]. Comparing Figure 7a,b, it should be clear that the CV curves of the cell with NSGPC-coated separator show more obvious oxidation/reduction peaks and higher peak currents than those of the cell with PP separator, indicating the stronger electrochemical reaction kinetics and improved active material utilization of the cell with NSGPC-coated separator [17]. Furthermore, the oxidation/ reduction peaks of CV profiles for the cell with NSGPC-coated separator are overlapped in respects of peak positions and peak currents in the great degree, again demonstrating the lower electrode polarization and better electrochemical reversibility than the cell with PP separator [46]. 

The discharge/charge voltage profiles of the Li-S batteries with PP and NSGPC-coated separators at 0.2 C are shown in Figure 7c,d (1 C = 1675 mA g^−1^), and the specific capacities are calculated by the weight of sulfur. Two representative discharge voltage plateaus and one charge voltage plateau are presented from both graphs, which accords well with the redox peaks showed in the CV curves (Figure 7a,b). However, the Li-S battery with NSGPC-coated separator shows smaller voltage gap and longer voltage plateaus in comparison to the Li-S battery with PP separator. Besides, along with the increasing number of cycles, the discharge voltage of the battery with PP separator decreases (Figure 7c), whereas the discharge voltage of the battery with NSGPC-coated separator remains almost the same (Figure 7d). These results obviously prove that the battery with NSGPC-coated separator has superior oxidation-reduction dynamics and reversibility of the electrode than the battery with PP separator.

**Figure 8:**
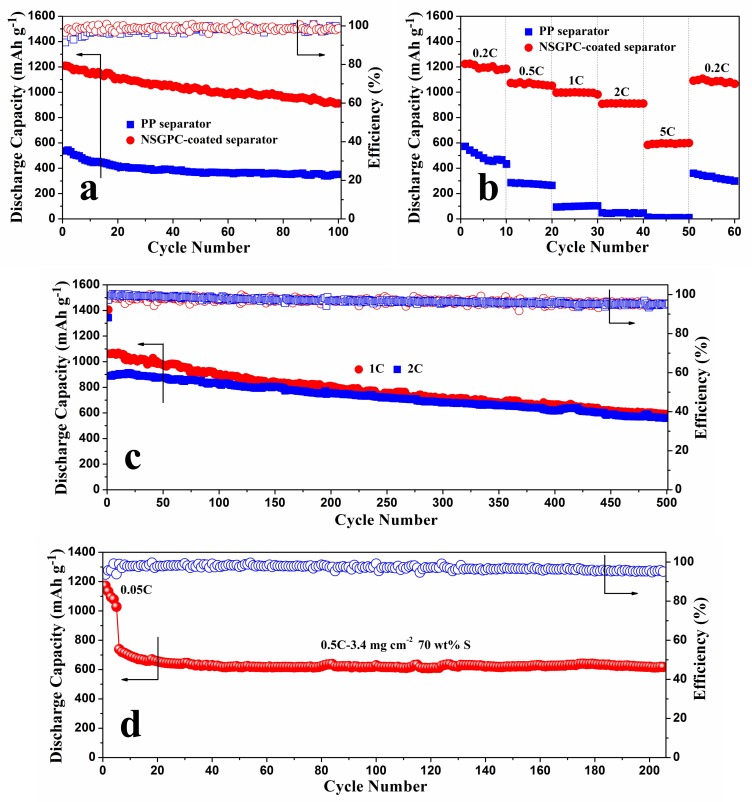
(**a**) Cyclic performances of the Li-S batteries with PP and NSGPC-coated separators at 0.2 C, (**b**) rate capabilities of the Li-S batteries with PP and NSGPC-coated separators at varied current density from 0.2 to 5 C, (**c**) long-range cyclic performance at 1 and 2 C for the Li-S batteries with NSGPC-coated separator, (**d**) cyclic performance at 0.5 C for the Li-S batteries with NSGPC-coated separator. The sulfur mass loading for (**a**–**c**) and (**d**) is about 1.3 and 3.4 mg cm^−2^, respectively.

The cycling performances for the above-mentioned batteries at 0.2 C are shown in Figure 8a. The Li-S battery with NSGPC-coated separator delivers an initial discharge capacity of 1208.6 mAh g^−1^ (with a coulombic efficiency of 96.6%). After 100 cycles, the identical battery still delivers a large reversible capacity of 911.7 mAh g^−1^ with a good capacity retention of 75.4%. In comparison, the traditional Li-S battery with PP separator exhibits an inferior initial discharge capacity of 536.4 mAh g^−1^ (with a coulombic efficiency of only 91.4%), and offers a reversible capacity of only 351.2 mAh g^−1^ after 100 cycles. The remarkable improvement of the electrochemical properties for the Li-S battery with NSGPC-coated separator is attributed to the efficient trapping of dissolved polysulfides within the porous matrix of the conductive NSGPC layer, which limits the notorious shuttle effect and thus improves the sulfur utilization.

The rate capabilities of the Li-S batteries with PP and NSGPC-coated separators were further investigated at various current rates from 0.2 to 5 C, as displayed in Figure 8b. For the battery with NSGPC-coated separator, after an initial discharge capacity of 1223.3 mAh g^−1^ at 0.2 C, the retention capacity reaches a final value of 1184.5 mAh g^−1^. When cycling at 0.5, 1, and 2 C, the reversible capacities remain at 1061.2, 997.4, and 910.3 mAh g^−1^, respectively. Even at a higher rate of 5 C, the battery still delivers a reversible capacity of 596.6 mAh g^−1^, exhibiting a favorable high rate performance. When the current is switched abruptly from 5 to 0.2 C, a large reversible capacity of 1066.5 mAh g^−1^ is still recovered, indicating an excellent redox stability of the battery with NSGPC-coated separator. However, the battery with PP separator suffers from dramatic capacity loss at the same increasing discharge/charge rates. In addition, Figure S4 shows the corresponding charge/discharge voltage profiles of the Li-S batteries with PP and NSGPC-coated separators for some selected cycles. We can clearly see that the battery with PP separator exhibits no distinct charge/discharge voltage platforms at 1 C. However, the battery with NSGPC-coated separator demonstrates two representative discharge voltage plateaus and one charge voltage plateau even at a high rate of 5 C. Such an outstanding rate performance of the battery with NSGPC-coated separator may benefit from the good electrical conductivity and the effective sequestration of the dissolution of polysulfides of NSGPC layer.

The long-range cyclic performance of the Li-S batteries with NSGPC-coated separator was also tested at higher current densities of 1 and 2 C, as shown in Figure 8c. After activating the batteries at 0.05 C for one cycle, the initial discharge capacities of 1061.6 and 890 mAh g^−1^ at 1 and 2 C are delivered, respectively, and the superior discharge capacities of 589.1 and 559.4 mAh g^−1^ are retained even after 500 cycles. Besides, the average coulombic efficiency is about 97%, and the corresponding capacity fade rates are as low as 0.089% and 0.074% per cycle, at 1 and 2 C, respectively.

The NSGPC-coated separator is also demonstrated to promote the property of a thick cathode, which shows the potential application for practical Li-S batteries. Here, we use a thick pure sulfur cathode with the sulfur content and mass loading of 70 wt. % and 3.4 mg cm^−2^, respectively. As displayed in Figure 8d, the cathode is firstly conducted at 0.05 C for 5 cycles to activate the battery, and an initial reversible capacity of 1171.7 mAh g^−1^ is delivered. The current is then switched from 0.05 to 0.5 C, the battery with NSGPC-coated separator exhibits an initial capacity of 738.6 mAh g^−1^ and also a reserved capacity of 617.7 mAh g^−1^ after 200 cycles at 0.5 C. The fading rate is only 0.082% of the initial capacity of 0.5 C per cycle, and the average coulombic efficiency is about 97.1%. Furthermore, the battery with NSGPC-coated separator always exhibits the obvious two-plateaux discharging behavior during the charge/discharge processes even using a thick sulfur cathode with high mass loading of 3.4 mg cm^−2^ (Figure S5). 

According to the above results, the large reversible capacity and excellent rate capability as well as long-term cycling life of the Li-S battery with NSGPC-coated separator can be largely attributed to the following virtues of the NSGPC: (i) the conductive NSGPC coating can serve as a second current collector to enhance the reaction activity during cycling, and thus improve the active sulfur utilization [23,24]; (ii) the existence of affluent micro-/mesopores not merely can inhibit the dissolution of polysulfides through physical adsorption, but also can facilitate electrolyte infiltration and provide fast transport channels for Li^+^ ions and electrons, and thus reutilize the intercepted active sulfur [31,44]; (iii) the in situ N and S co-doping could remarkably enhance the electric conductivity of carbon and further anchor lithium polysulfides through strong chemical interaction between Li^+^ and doped N atoms (Li-N) as well as the polysulfide anions and doped S atoms (S-S) [31,32,33,34,35].

To further research the influence of the NSGPC-coated separator on immobilizing polysulfides, the Li-S batteries with PP and NSGPC-coated separators after 100 cycles at 0.2 C were disassembled in an Ar-filled glove box. The cycled NSGPC-coated separator was first thoroughly washed with DME/DOL solvent for SEM analysis. Figure S6 displays the SEM images and corresponding elemental mappings of NSGPC-coated separator before cycles and after 100 cycles at 0.2 C. The elemental mappings of NSGPC-coated separator before cycles show that the sulfur signals are probed, this is because the pristine NSGPC samples possess some amount of sulfur (Figure 5 and Figure S2). Nevertheless, after 100 cycles at 0.2 C, a homogeneous and strong sulfur signals can be easily detected in the elemental mappings of cycled NSGPC-coated separator (Figure S6d). This phenomenon reveals the effectiveness of the NSGPC coating in confining and reutilizing the polysulfides on the cathode side rather than permitting them to migrate freely through the separator to the Li anode. Furthermore, the elemental mappings of the back of NSGPC-coated separator (facing to the Li anode) after 100 cycles at 0.2 C show negligible sulfur signals (Figure S7), further verifying the above view.

**Figure 9:**
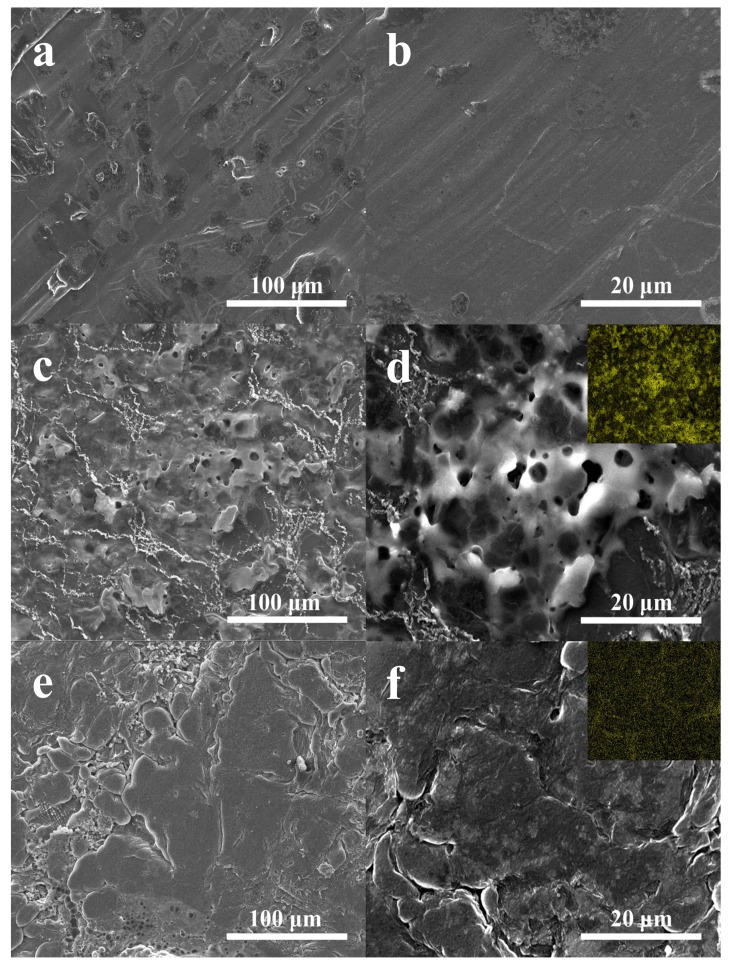
SEM images of (**a**,**b**) the fresh Li anode, and the Li anodes in the batteries with (**c**,**d**) PP and (**e**,**f**) NSGPC-coated separators after 100 cycles at 0.2 C, the insets in d and f are the corresponding elemental maps of sulfur.

The surface morphology analysis of the cycled Li anodes from the above-mentioned batteries with PP and NSGPC-coated separators was also carried out by SEM. The surface of the Li anode before cycling is very glossy and flat (Figure 9a,b). Nevertheless, as shown in Figure 9c,d, the cycled Li anode of the battery with PP separator exhibits a highly coarse and intense corrosive surface, and the elemental map (inset of Figure 9d) also displays massive sulfur signal, which can be caused by the dissolved polysulfides across to anode region to react with Li anode and the subsequent deposition of side reactions products (Li_2_S_2_/Li_2_S) during cycling [31,47]. In contrast, the cycled Li anode of the battery with NSGPC-coated separator shows relatively glossy surface with less corrosion (Figure 9e,f) and faint sulfur signal (inset of Figure 9f). All of the above results strongly suggest that the polysulfides shuttle and relevant side reactions were greatly suppressed by the NSGPC coating, thus enhancing the active sulfur utilization and prolonging the life expectancy of Li anode.

In order to research the effect of the modified separator on the electrode reaction resistance, the EIS measurements of the fresh Li-S batteries with PP and NSGPC-coated separators were performed (Figure S8). The Nyquist plots of both batteries consist of a quasi-semicircle in the high-to-medium frequency area (associating with the charge transfer resistance R_ct_) and an oblique line at the low-frequency area (corresponding to the Warburg impedance W_o_) [23]. The R_ct_ value of the battery with NSGPC-coated separator (35.8 Ω) is about three times lower than that of the battery with PP separator (122.7 Ω). This is due to the high conductivity of NSGPC coating that can remarkably enhance the interfacial charge transfer of the electrode [47]. Moreover, the value of W_o_ for the battery with NSGPC-coated separator (4.4 Ω) is far lower than that of the battery with PP separator (147.3 Ω), indicating that the NSGPC coating can provide an excellent Li^+^ ion diffusion pathway within the electrode [48,49].

## 4. Conclusions

In summary, sandwich-type NSGPC has been successfully fabricated via a combined hydrothermal and KOH activation process and subsequently employed to modify the commercial PP separator in Li-S batteries. Benefiting from the particular physical properties and chemical components of NSGPC, the as-synthesized NSGPC-coated separator for Li-S batteries not only can serve as an upper current collector to offer rapid Li^+^ ions and electrons transport pathways, but also can restrict the dissolution of polysulfides to alleviate the shuttle effect by combining the physical absorption and strong chemical adsorption. The XPS studies reveal that this strong chemical interaction arises from Li-N and S-S interactions between polysulfides and codoped N/S atoms. Thanks to these functional virtues, the Li-S batteries with NSGPC-coated separator demonstrate a superior high initial reversible capacity of 1208.6 mAh g^−1^ at 0.2 C and low capacity fading of 0.074% per cycle for over 500 cycles at 2 C. Besides, the Li-S batteries with this functional composite separator and an easy-designed pure sulfur cathode with an increased mass loading of 3.4 mg cm^−2^ (70 wt. % sulfur) deliver a reversible capacity of 617.7 mAh g^−1^ after 200 cycles at 0.5 C. The results of this research would be extremely valuable for the reasonable design of multifunctional composite separators with a pivotal role in the commercialized applications of high-performance Li-S batteries.

## Supplementary Materials

The following are available online at http://www.mdpi.com/2079-4991/8/4/191/s1, Figure S1: SEM images of pure G (a,b), TEM and HRTEM images of pure G (c,d), Figure S2: STEM image of NSGPC (a), corresponding elemental maps of carbon (b), oxygen (c), nitrogen (d), and sulfur (e), Figure S3: Digital photograph of Li_2_S_4_ solution and after adding NSGPC, Figure S4: Discharge/charge voltage profiles of the Li-S batteries with (a) PP and (b) NSGPC-coated separators at various current rates. The sulfur mass loading of the simple cathodes was about 1.3 mg cm^−2^, Figure S5: Discharge/charge voltage profiles of the Li-S batteries with NSGPC-coated separator at various cycles. The sulfur mass loading of the simple cathodes was about 3.4 mg cm^−2^, Figure S6: SEM images and corresponding elemental mappings of NSGPC-coated separator (a,b) before cycles and (c,d) after 100 cycles at 0.2 C, Figure S7: SEM image (a) and corresponding elemental mappings (b) of the back of NSGPC-coated separator (facing to the Li anode) after 100 cycles at 0.2 C, Figure S8: EIS curves of the fresh Li-S batteries with PP and NSGPC-coated separators, and the inset is equivalent circuit model.
