# Supplementary material for: Sandwich-Type Nitrogen and Sulfur Codoped Graphene-Backboned Porous Carbon Coated Separator for High Performance Lithium-Sulfur Batteries
Source: Nanomaterials (Basel). 2018 Mar 26;8(4):191. doi: 10.3390/nano8040191 (PMC5923521; doi:10.3390/nano8040191)
Supplement: Supplementary file 1 [file nanomaterials-08-00191-s001.pdf]

Supplementary Files

Sandwich-Type Nitrogen and Sulfur Codoped Graphene-Backboned Porous Carbon Coated Separator for High Performance Lithium-Sulfur Batteries

Feng Chen [1], Lulu Ma [1], Jiangang Ren [1], Xinyu Luo [2], Bibo Liu [1,*] and Xiangyang Zhou [2,*]

[1] School of Resource and Environment, Henan University of Engineering, No. 1, Xianghe Road, Xinzheng, Zhengzhou, 451191, China; chenfeng871588@163.com (F.C.); malulu1001@163.com (L.M.); renjiangang2005@126.com (J.R.)

[2] School of Metallurgy and Environment, Central South University, Lushan South Street 932, Yuelu District, Changsha, 410083, China; 17307484092@163.com

* Correspondence: liubb2008@126.com (B.L.); hncsyjy308@163.com (X.Z.); Tel.: +86-037162508218 (B.L.); +86-073188836329 (X.Z.)

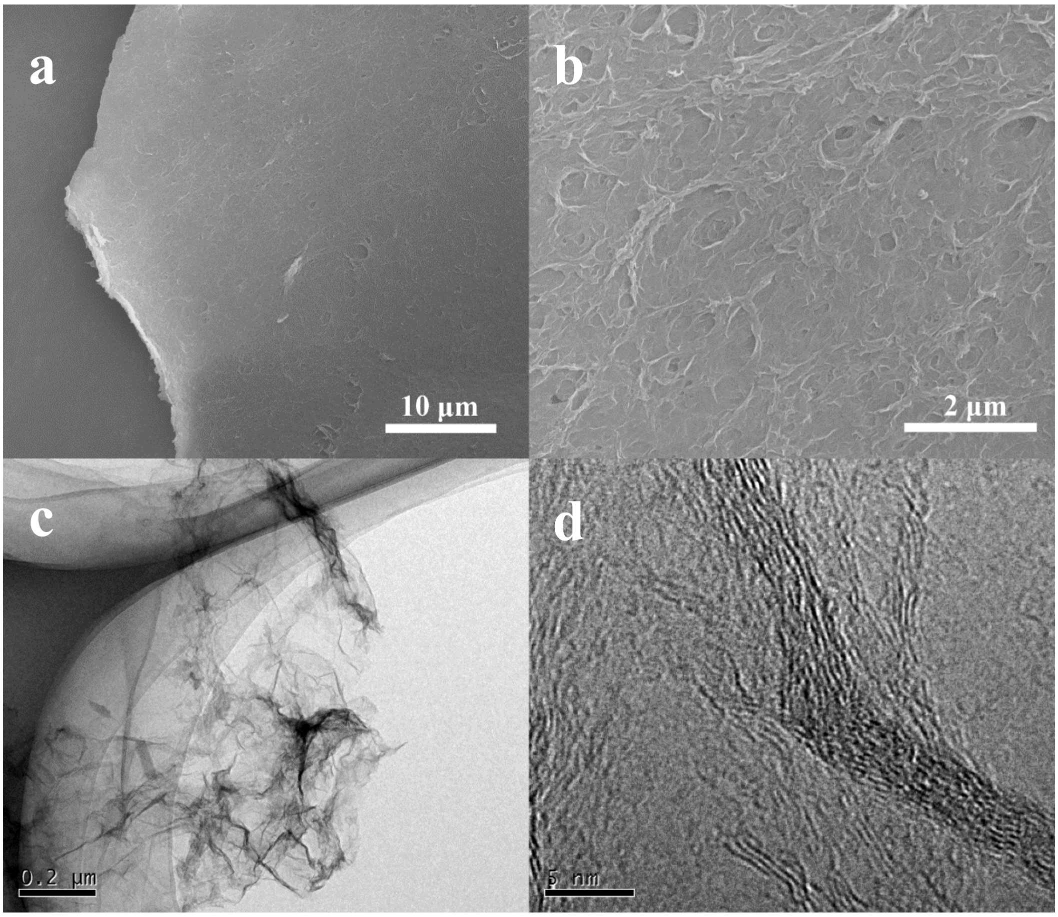

Figure S1. SEM images of pure G (a-b), TEM and HRTEM images of pure G (c-d).

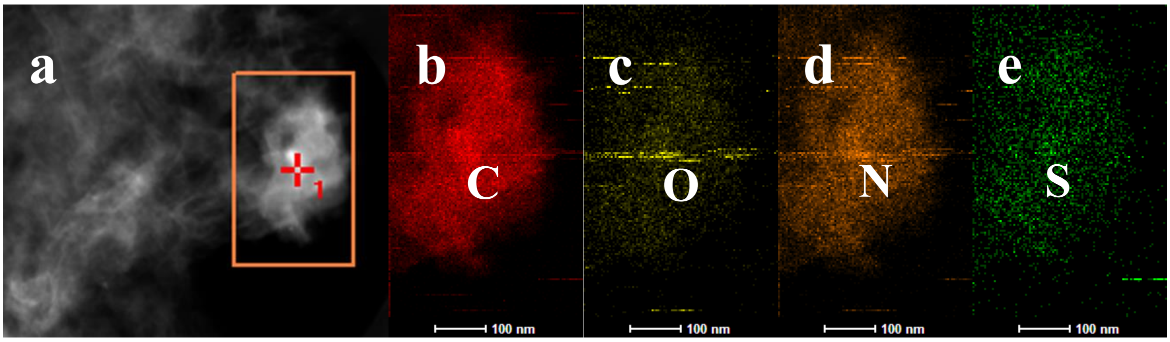

Figure S2. STEM image of NSGPC (a), corresponding elemental maps of carbon (b), oxygen (c), nitrogen (d), and sulfur (e).

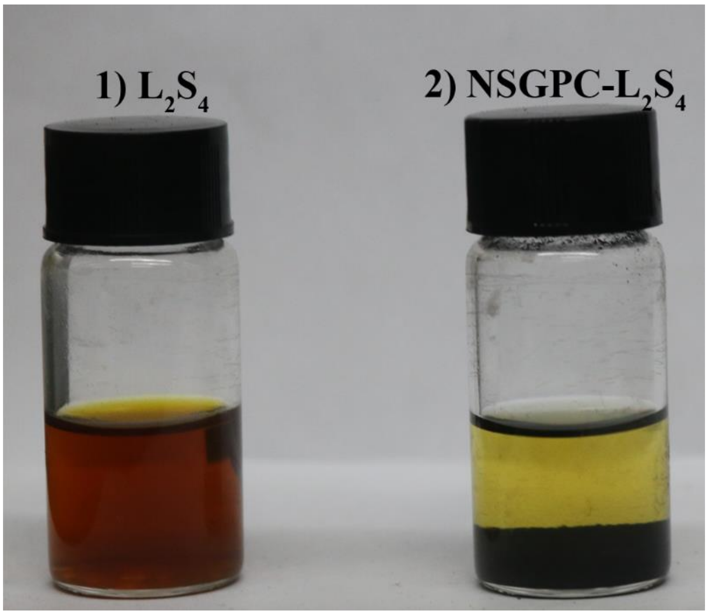

Figure S3. Digital photograph of Li_2S_4 solution and after adding NSGPC.

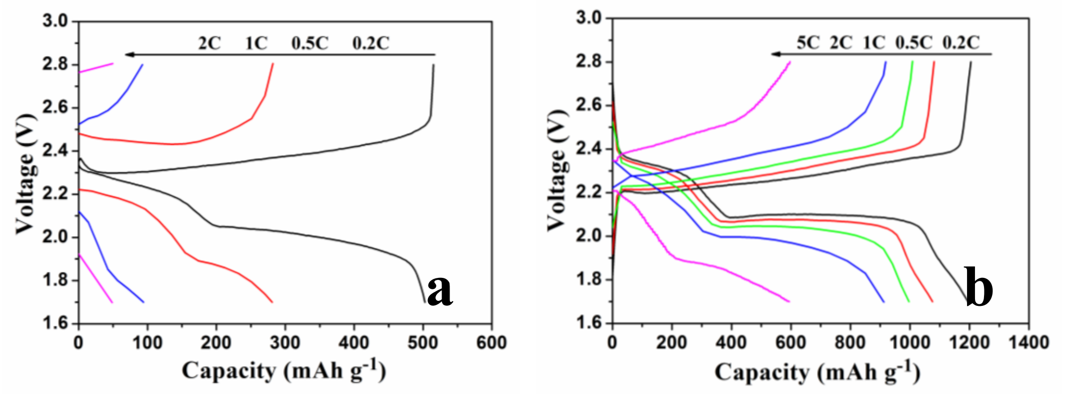

Figure S4. Discharge/charge voltage profiles of the Li-S batteries with (a) PP and (b) NSGPC-coated separators at various current rates. The sulfur mass loading of the simple cathodes was about 1.3 mg cm^{-2}.

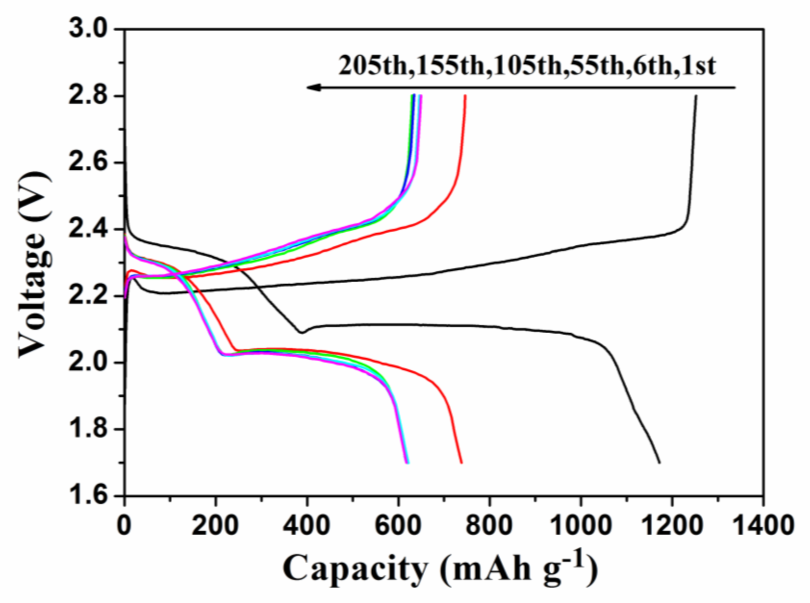

Figure S5. Discharge/charge voltage profiles of the Li-S batteries with NSGPC-coated separator at various cycles. The sulfur mass loading of the simple cathodes was about 3.4 mg cm^{-2}.

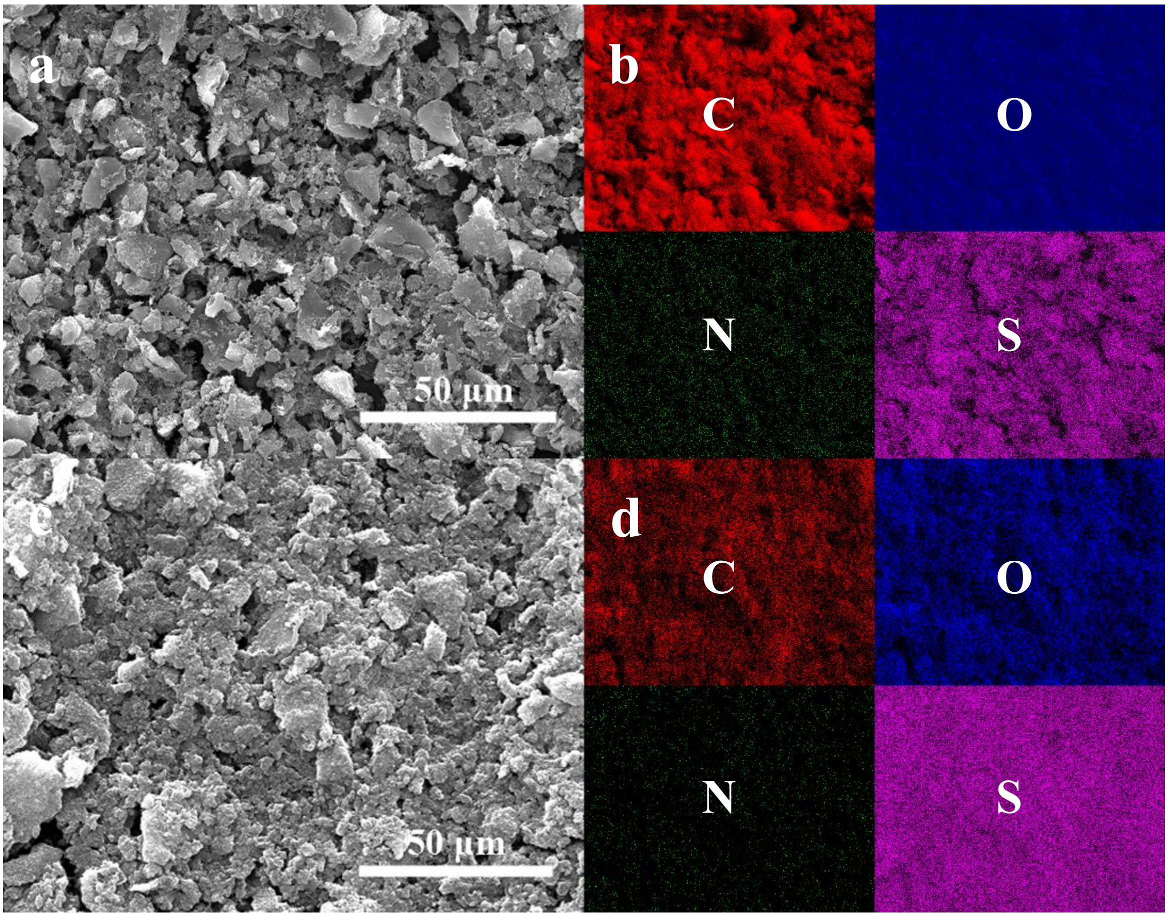

Figure S6. SEM images and corresponding elemental mappings of NSGPC-coated separator (a,b) before cycles and (c,d) after 100 cycles at 0.2 C.

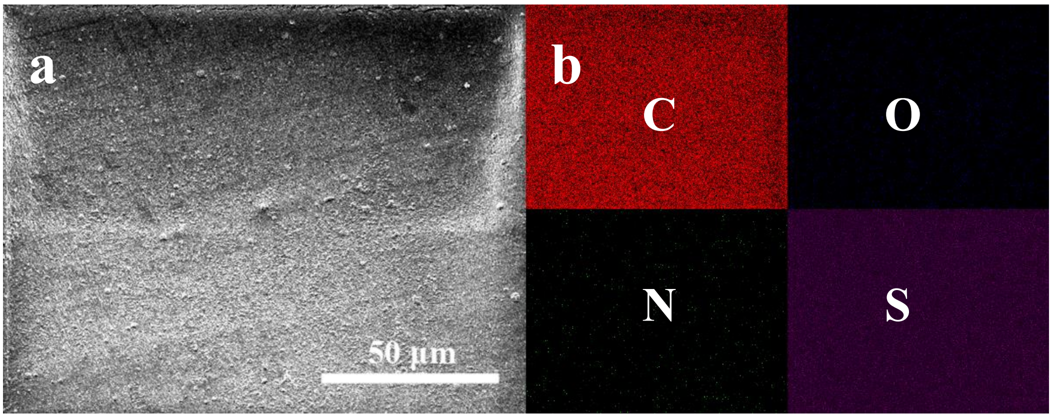

Figure S7. SEM image (a) and corresponding elemental mappings (b) of the back of NSGPC-coated separator (facing to the Li anode) after 100 cycles at 0.2 C.

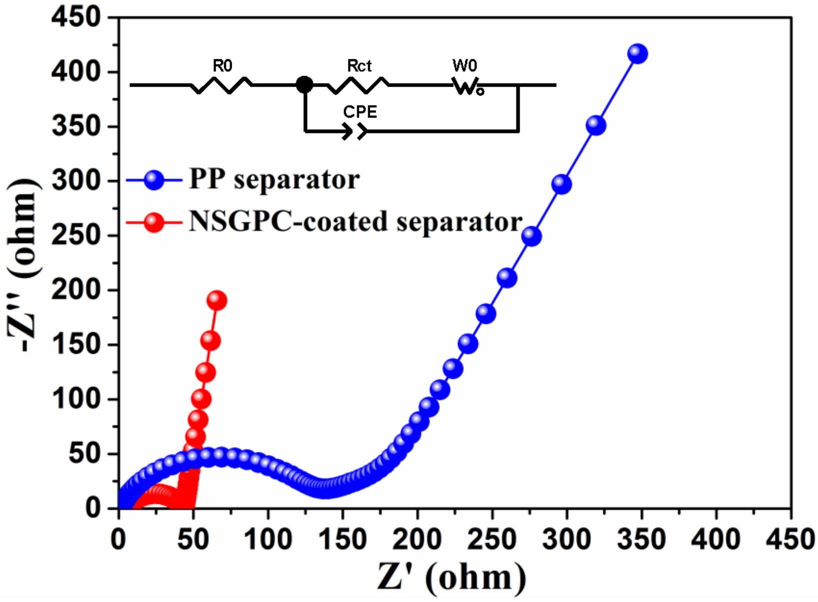

Figure S8. EIS curves of the fresh Li-S batteries with PP and NSGPC- coated separators, and the inset is equivalent circuit model.
